# Phylogenetic insight of Nonribosomal peptide synthetases (NRPS) Adenylate domain in Antibacterial potential Streptomyces BDUSMP 02 isolated from Pitchavaram Mangrove

**DOI:** 10.6026/97320630015412

**Published:** 2019-06-15

**Authors:** Periyasamy Sivalingam, Manickam Muthuselvam, John Pote, Kandasamy Prabakar

**Affiliations:** 1 Department F.A. Forel for Environmental and Aquatic Sciences and Institute of Environmental Sciences, School of Earth and Environmental Sciences, Faculty of Science, University of Geneva, Uni Carl Vogt, 66 Boulevard Carl-Vogt, CH-1211 Geneva 4, Switzerland; 2 Department of Biotechnology and Genetic Engineering, Bharathidasan University, Tiruchirappalli-620024, Tamil Nadu, India; 3 Postgraduate and Research Department of Zoology, Jamal Mohamed College, Tiruchirappalli-620020, Tamil Nadu, India

**Keywords:** Mangrove, Streptomyces, NRPS, adenylate domain, Phylogeny

## Abstract

Identification of gene clusters in Streptomyces holds promise for the discovery of regulatory pathways linked to bioactive metabolites. We isolated a broad-spectrum antibacterial potential Streptomyces sp BDUSMP 02 from mangrove sediment. We further found a distinct of phylogeny pattern for NRPS A-domain in the Streptomyces sp BDUSMP 02. The result suggests that Streptomyces sp BDUSMP 02 has the potential to produce a new type of antibacterial compounds belonging to NRPS type.

## Background

In the last five decades, natural compounds produced by actinobacteria have been enormously utilized to develop most of the common antibiotics commercialized by pharmaceutical industries 1. Given this, isolation of actinomycetes from unexplored marine environment has been attracted particular attention due to their structural diversity and distinct bioactivities of secondary metabolites produced by them 2. In evidence, Salinispora comes under the genus Actinomycete was first isolated from ocean sediments 3. Mangrove forests are located in the tidal zones in tropical and subtropical regions 4. Bissett et al. (2007) reported that mangrove sediments are known to contain high organic content, which favour the rapid development of species diversity corresponding to environmental variation 5. The exploitation of mangrove actinomycetes for bioactive compounds has been increased dramatically 6-8. Streptomyces sp isolated from mangrove ecosystem have been able to grow in freshwater, brackish water and seawater which suggest that they are adapted to various environmental conditions due to the water current 7. Besides, it could be a starting point to study the evolution of gene clusters responsible for the biosynthesis of novel antibiotic because of their adaptation to extraordinarily salty and marshy condition 7.

It is evident that gene clusters in Streptomyces likely to encode natural product biosynthetic pathways in sequenced microbial genomes 9. The biosynthetic potential of different strains isolated from various sources can be approximated by the detection of the genes involved in the synthesis of secondary metabolites such as those for a polyketide synthase (PKS) and non-ribosomal peptide synthetase (NRPS). Non-ribosomal peptide synthetases (NRPSs) are megaenzymes usually with a multimodular structure, which catalyze the non-ribosomal assembly of peptides from proteinogenic and non-proteinogenic amino acids 10, 11. Schwarzer and Marahiel, (2001) reported that an NRPS module usually contains an adenylation domain (A-domain), a peptidyl carrier protein domain (PCP-domain), and a condensation domain (C-domain) 12. There are eight core motifs have been postulated in A domain of NRPS including A1-A8 (A1, LKAGxAYVPID; A2, LAYxxYTSGTTGxPKG; A3, FDxS; A4, NxYGPTE; A5, GELxLxGxGLARGYW; A6, YKTGDQ; A7, GRxDxQVKIRGx; A8, NGKIDR) and they are highly conserved 13. Komaki and Harayama (2006) reported that DNA sequence based on these genes could be used to predict the chemical nature of compounds 14. The biological functions of NRPS via synthesized compounds associated with the chemical nature of peptide, which is correlated with the gene sequence 11. Therefore it is crucial to study the phylogenic insight of NRPS in the potential actinomycete would provide new opportunities for drug discovery.

## Methodology

### Isolation and identification of Actinomycetes:

Soil samples were collected from Mangrove sediment of Pitchavaram (Latitude of 11.4' N-Longitude of 79.8' E), Tamil Nadu, India, in sterile airlock polythene bags and transported to the laboratory according to a previously described method 6. One gram of air-dried each spot soil samples was added to a 9 ml of sterile water and subjected to selective pretreatment of dry heat at 56 °C for 10 min to effectively increase the number of myceliumforming actinomycetes relative to the non-actinomycetal heterotrophic microbial flora. After that, the samples were vigorously shaken and further diluted up to 10 -6 in sterile water. 100 µl of each diluted sample was inoculated by spreading with a sterile glass rod onto humic acid-vitamin B agar (HV) medium (Hayakawa and Ohara, 1987) supplemented with antibiotics of cycloheximide (40 µg/ml), nystatin (30 µg/ml) and nalidixic acid (10 µg/ml) after autoclave to inhibit the fungal and nonfilamentous bacterial growth. The inoculated plates were incubated at 30 °C for ten days or until appearance of colonies with a tough leathery texture, dry or folded appearance, and branching filaments with or without aerial mycelia.

### Antibacterial activity:

Lyophilized cultures of Escherichia coli (MTCC 1687); Vibrio cholerae (MTCC 3906); Proteus mirabilis (MTCC 425); Klebsiella pneumoniae (MTCC3384); Staphylococcus aureus (MTCC 3160); Salmonella typhi (MTCC3231) was obtained from Microbial Type Culture Collection, IMTECH, India. All test strains were cultured at 37 °C in Luria broth or on Luria agar. Antibacterial activity of cell-free supernatant was determined using the disc diffusion method. 100 µl of 8 hrs old culture broth of the test organisms were spread over on the surface of the Luria agar plate using a sterile cotton swab. After that, 60 µl of cell-free supernatant was added into sterile standard discs (Himedia, India) and incubated for 18 to 24 hrs at 37 °C. After that the clear incubation zone of inhibition (ZOI) was measured to evaluate the antimicrobial activity of actinomycetes isolate. Dimethyl sulphoxide (DMSO) was used as control.

### NRPSs amplification, cloning, and sequencing

The NRPSs gene was amplified from genomic DNA of Streptomyces sp BDUSMP 02 using the previously described degenerate primers ADEdom 5'- CCA ACS GGC NNN CCS AAG GGC GT 3' and ADEdom 3' - ACC CTC SGT SGG SCC GTA 3' 15. A 450- bp fragment encoding the adenylation domain of NRPS was amplified. The PCR was performed in a total volume of 50 µl containing one µM of each primer, 1µl of extracted DNA, 23 µl of sterile distilled H2O, and 10% (v/v) of DMSO to the final volume in PCR premix (Emerald, Takara, Japan). Amplifications were performed in a thermal cycler (Eppendorf, Germany). Polymerase chain reaction conditions were as follows: 5 min at 95 °C followed by 40 cycles of 1 min at 94 °C, 1 min at 60 °C and 1.5 min at 72 °C followed by a 10-min final extension step at 72 °C. The purified PCR product was ligated pGEM^®^ -T Vector according to the manufacturer's instructions. Plasmid DNA from the transformants was isolated using the HiYield TM Plasmid Mini Kit (RBC, Korea). To confirm the cloned product, PCR was performed again using the primer pairs of T7 Promoter 5' TAA TAC GAC TCA CTA TAG GG 3' and SP6 Promoter 5' GAT TTA GGT GAC ACT ATA G 3'. The PCR was performed in a total volume of 50 µl containing one µM of each primer, 2 µl of extracted plasmid DNA, 23 µl of sterile distilled H2O, and 10% (v/v) of DMSO to the final volume in PCR premix (Emerald, Takara, Japan). Amplifications were performed in a thermal cycler (Eppendorf, Germany). Polymerase chain reaction conditions were as follows: 5 min at 95°C followed by 35 cycles of 1 min at 94 °C, 1 min at 54 °C and 1.5 min at 72 °C followed by a 10- min final extension step at 72°C. The DNA sequencing was performed on an ABI 310 automatic DNA sequencer (Applied Biosystems) using the SP6 and T7 promoters primers.

### Phylogenetic analysis of NRPS Adenylate domain

BLAST network services at the NCBI were used to analyze the resulting NRPSs gene sequence 16. Multiple alignments were performed using CLUSTAL_X version 1.8 17. The phylogenetic tree was inferred Neighbor-joining method using MEGA 6.0 software package 18. The unrooted phylogenetic tree topology was evaluated by using the bootstrap resamplings method with 1000 replicates 19.

## Results and Discussion:

### Isolation and characterization of Mangrove Actinomycete:

The results of morphological, physiological and biochemical characteristics of strain BDUSMP 02 are shown in (Table 1). The cell wall of the strain found to contain LL-diaminopimelic acid (chemotype I), which is characteristic for the genus Streptomyces. Phylogenetic analysis of the 16S rRNA gene sequence (1388 bp) of strain BDUSMP 02 revealed that the isolate belongs to the genus Streptomyces. The 16S rDNA sequence has been deposited in the GenBank database under Accession No. KF918272.1. Based on morphological, physiological, biochemical characterization and 16S rDNA sequence analysis, the isolate was named as Streptomyces sp. BDUSMP 02.

### Antibacterial activity:

The isolate displayed significant antibacterial activities against test strains. The zone of inhibition was 15 mm, 14 mm, 18 mm, 18 mm, 12 mm and 10 mm for Escherichia coli (MTCC 1687), Vibrio cholerae (MTCC 3906), Klebsiella pneumoniae (MTCC3384), Proteus mirabilis (MTCC 425), Salmonella typhi (MTCC3231) and Staphylococcus aureus (MTCC 3160), respectively. In marine environments, it is noteworthy that mangrove sediments are known to have novel actinomycetes 6. It has been documented that new bioactive compounds have been obtained from mangrove actinomycetes 20. In agreement with those previous reports, the results presented in this study denoted that bioactive secondary metabolite production by the mangrove sediment actinomycete and its gene clusters responsible for the biosynthesis could be at a later stage taken into the molecular biology of natural product research. 

### NRPSs gene adenylation domain (A-domain):

**Figure 1:**
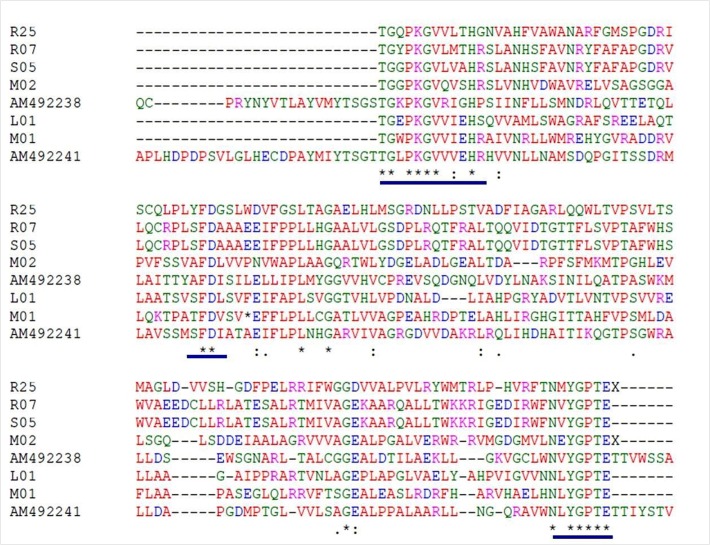
Amino acid sequence alignment of the patterns for the identification of NRPS A-domain conserved motifs. R25: Streptomyces sp BDUSMP R25; R07: Streptomyces sp BDUSMP R07; S05: Streptomyces sp BDUSMP S05; M02: Streptomyces sp BDUSMP 02; L01: Streptomyces sp BDUSMP L01; M01: Streptomyces sp BDUSMP 01.

Streptomyces sp. BDUSMP 02 non-ribosomal peptide synthetase gene, partial cds, has been deposited in GenBank database under Accession No. KJ598809.1. The resulting amino acid sequences corresponding to their nucleotide sequences of amplified NRPS Adomain showed conserved motif, as shown in Figure 1. There are three core motifs in the amplified 450 bp fragments of A-domain identified including A2, TGxPKGV, A3, FD and A4, NxYGPTE.

NRPS A domain was best matched with the previously reported Streptomyces. The resulting amino acid sequences shared low similarities with those available in databanks. Liu et al. (2019) reported that Streptomyces isolated from mangrove sediment harbouring NRPS genes which involved in the synthesis of the antibacterial compound. Secondary metabolite production in Streptomyces is growth dependent and involves the expression of physically clustered regulatory and biosynthetic genes by a tightly regulated mechanism 21. Similarly, in the present study, biosynthetic NRPS gene sequences provided valuable genomicbased information in parallel with the antimicrobial activity of isolate. Our results thus proved the presence of NRPS genes in support of the bioassay-guided analysis for antibacterial activity.

### Phylogenetic analysis of NRPSs A-domain:

**Figure 2:**
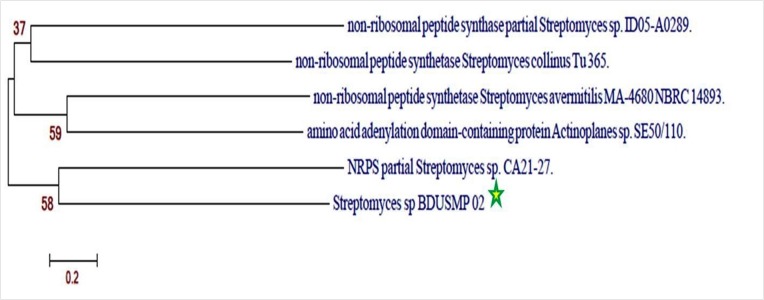
NRPS Adenylate domain phylogenetic analysis of Streptomyces sp BDUSMP 02. The evolutionary history of Streptomyces sp BDUSMP 02 was inferred using the Neighbor-Joining method. The percentage of replicate trees in which the associated taxa clustered together in the bootstrap test (1000 replicates) is shown next to the branches. The evolutionary distances were computed using the Poisson correction method and are in the units of the number of amino acid substitutions per site. All positions containing gaps and missing data were eliminated. Evolutionary analyses were conducted in MEGA6.

Figure 2 presents the phylogenetic tree of Streptomyces sp BDUSMP 02 based on NRPS A domain amino acid sequence. The NRPS A - domain amino acid sequence of the isolate Streptomyces sp BDUSMP 02 showed a less identity to the sequences from various Streptomyces sp. A good agreement between bioassay-guided identification antibacterial properties this isolate had functional NRPS genes in their putative gene cluster responsible for the synthesis of antibacterial compounds. Interestingly the strain NRPS A - domain shared similarity with Streptomyces avermilities. It is therefore desirable to isolate the secondary metabolite with antibacterial properties to relate with its functional genes.

## Conclusion

We describe a Streptomyces sp. from mangrove environment as a promising source of novel antibacterial compounds. There is increasing interest in the characterization of gene clusters, which mainly contain NRPS, PKS and NRPS/PKS in addition to culturedependent experimentation for distinct bioactivities. We found NRPS adenylate domain from the potential isolate, which can be further explored for the drug discovery using a genome mining approach.

## Conflict of Interest

The authors declare that there is no conflict of interests regarding the publication of this paper.

## Figures and Tables

**Table 1:** Cultural, physiological and biochemical characteristics features of the isolated Streptomyces sp BDUSMP 02 from mangrove sediment.

Characteristics	Streptomyces sp BDUSMP 02							
Features	ISP2	ISP3		ISP4		ISP5	ISP6	ISP7
Growth	Good	Good		Good		Moderate	Poor	Moderate
Sporulation	Good	Good		Good		Moderate	Poor	Moderate
Colour of aerial mycelium	Grayish brown	Grayish white		Gray		Gray	Light yellow	Gray
Colour of substrate mycelium	Brown	Light Gray		light brown		Light brown	Yellow	Blackish Gray
Diffusible Pigment	no	no		no		no	no	no
Biochemical								
Gram staining						Positive		
Citrate utilization						Positive		
Methyl Red						Negative		
Voges-Proskauer						Negative		
H2S production						Negative		
Nitrate reduction test						Positive		
Catalase test						Negative		
Urea hydrolysis						Positive		
Gelatin hydrolysis						Negative		
Starch hydrolysis						Negative		
Utilization of carbon								
Arabinose						-		
Dextrose						+		
Fructose						+		
Inosittol						-		
Lactose						+		
Mannitol						+		
Maltose						-		
Sucrose						+		
Xylose						-		
Growth on sole nitrogen source								
Alanine						+		
Arginine						+		
Asparagine						+		
Cysteine						-		
Methionine						-		
Phenylalanine						-		
Effect of Temperature on growth								
T°C	25		28		32	37	40	45
	(++)		(+++)		(++)	(+)	(+)	(-)
Effect of Ph on growth								
pH	4		6		7	8	9	10
	-		(+)		(+++)	(+)	(-)	(-)
Effect of NaCl concentration on growth								
	0		3		5	7	9	11
%	(+)		(+)		(+)	(+)	(+)	(-)
